# Bioinformatic analysis of an unusual gene-enzyme relationship in the arginine biosynthetic pathway among marine gamma proteobacteria: implications concerning the formation of N-acetylated intermediates in prokaryotes

**DOI:** 10.1186/1471-2164-7-4

**Published:** 2006-01-12

**Authors:** Ying Xu, Nicolas Glansdorff, Bernard Labedan

**Affiliations:** 1 Marine Sciences Research Center, State University of New York at Stony Brook, Stony Brook, New York 11794-5000, USA; 2 Microbiology and Genetics, Vrije Universiteit Brussel, Pleinlaan 2, B-1050 Brussels, Belgium; 3 Institut de Génétique et Microbiologie, CNRS UMR 8621, Université Paris Sud, Bâtiment 400, 91405 Orsay Cedex, France

## Abstract

**Background:**

The N-acetylation of L-glutamate is regarded as a universal metabolic strategy to commit glutamate towards arginine biosynthesis. Until recently, this reaction was thought to be catalyzed by either of two enzymes: (i) the classical N-acetylglutamate synthase (NAGS, gene *argA*) first characterized in *Escherichia coli *and *Pseudomonas aeruginosa *several decades ago and also present in vertebrates, or (ii) the bifunctional version of ornithine acetyltransferase (OAT, gene *argJ*) present in Bacteria, Archaea and many Eukaryotes. This paper focuses on a new and surprising aspect of glutamate acetylation. We recently showed that in *Moritella abyssi *and *M. profunda*, two marine gamma proteobacteria, the gene for the last enzyme in arginine biosynthesis (*argH*) is fused to a short sequence that corresponds to the C-terminal, N-acetyltransferase-encoding domain of NAGS and is able to complement an *argA *mutant of *E. coli*. Very recently, other authors identified in *Mycobacterium tuberculosis *an independent gene corresponding to this short C-terminal domain and coding for a new type of NAGS. We have investigated the two prokaryotic Domains for patterns of gene-enzyme relationships in the first committed step of arginine biosynthesis.

**Results:**

The *argH-A *fusion, designated *argH(A)*, and discovered in *Moritella *was found to be present in (and confined to) marine gamma proteobacteria of the *Alteromonas- *and *Vibrio-*like group. Most of them have a classical NAGS with the exception of *Idiomarina loihiensis *and *Pseudoalteromonas haloplanktis *which nevertheless can grow in the absence of arginine and therefore appear to rely on the *arg(A) *sequence for arginine biosynthesis. Screening prokaryotic genomes for virtual *argH-X *'fusions' where *X *stands for a homologue of *arg(A)*, we retrieved a large number of Bacteria and several Archaea, all of them devoid of a classical NAGS. In the case of *Thermus thermophilus *and *Deinococcus radiodurans*, the *arg(A*)-like sequence clusters with *argH *in an operon-like fashion. In this group of sequences, we find the short novel NAGS of the type identified in *M. tuberculosis*. Among these organisms, at least *Thermus*, *Mycobacterium *and *Streptomyces *species appear to rely on this short NAGS version for arginine biosynthesis.

**Conclusion:**

The gene-enzyme relationship for the first committed step of arginine biosynthesis should now be considered in a new perspective. In addition to bifunctional OAT, nature appears to implement at least three alternatives for the acetylation of glutamate. It is possible to propose evolutionary relationships between them starting from the same ancestral N-acetyltransferase domain. In *M. tuberculosis *and many other bacteria, this domain evolved as an independent enzyme, whereas it fused either with a carbamate kinase fold to give the classical NAGS (as in *E. coli) *or with *argH *as in marine gamma proteobacteria. Moreover, there is an urgent need to clarify the current nomenclature since the same gene name *argA *has been used to designate structurally different entities. Clarifying the confusion would help to prevent erroneous genomic annotation.

## Background

**Figure 1:**
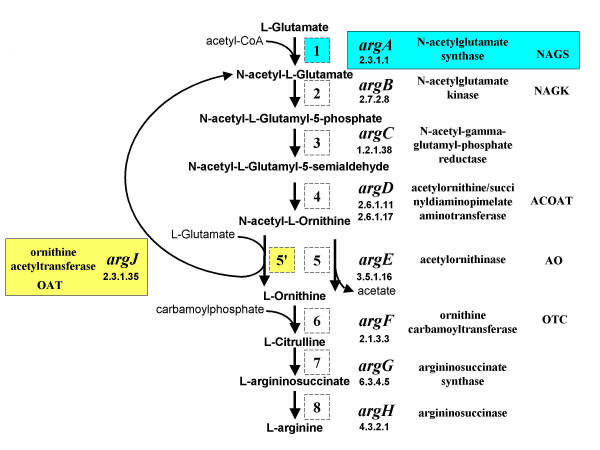
**The *de novo *arginine biosynthetic pathway**. Outline of the pathway; for each step (labeled by a number in a square) the following features are indicated: gene name, EC number, enzyme name and its abbreviation.

Until recently the *de novo *arginine biosynthetic pathway (Figs. 1A and 1B) was thought to conform to a simple type of one gene-one enzyme relationship even if it had been known for a long time that the fifth step, the conversion of *N*-α-acetyl-L-ornithine into L-ornithine, could be catalyzed by different enzymes. An ornithine acetyltransferase (OAT, ArgJ; EC 2.3.1.35) recycles the transfer of the acetyl group from acetylornithine to glutamate in most organisms (Bacteria, Archaea and Eukaryotes), whereas an acetylornithinase (AO; ArgE; EC 3.5.1.16) splits the acetyl group from acetylornithine in enteric and vibrio-like bacteria as well as in *Xylella fastidiosa, Xanthomonas campestris, Myxococcus xanthus *and, possibly, the crenarchaeon *Sulfolobus solfataricus *(see [1] and [2] for reviews).

Three findings concerning the status of acetylated intermediates in arginine synthesis and the enzymes involved in their genesis have rendered this picture more intricate. (i) In *X. campestris *and *Bacteroides fragilis*, an acetylornithine carbamoyltransferase was found to replace the canonical ornithine carbamoyltransferase (OTC; ArgF; EC 2.1.3.3) [3,4]; (ii) In *Bacillus stearothermophilus*, and *Thermotoga neapolitana*, OAT is bifunctional: in addition to recycling the acetyl group it also synthesizes N-acetylglutamate directly from acetylCoA and glutamate, the reaction catalyzed by N-acetylglutamate synthase (NAGS; ArgA; EC 2.3.1.1) [5]. Since the genomes of their closely related organisms, *B. subtilis *and *T. maritima *respectively, appear to lack a NAGS, the question arises whether some organisms use a bifunctional OAT instead of a NAGS to synthesize acetylglutamate [[5] and references therein, [6]]. (iii) Two novel species of marine gamma proteobacteria belonging to the genus *Moritella*, display an unusual gene structure for argininosuccinase (ArgH; EC 4.3.2.1), the last enzyme of the pathway: *argH *gene is extended by a ± 170 codon-long stretch which can complement *E. coli *mutants deficient in NAGS [7,8]. This new gene was called *argH(A)*. The *(A) *sequence is homologous to the C-terminal domain of *Escherichia coli *NAGS: this domain contains an acetylCoA N-acyltransferase fold (see [1]) whereas the N-terminal domain of *E. coli *NAGS presents striking similarities with the carbamate kinase-like domain of N-acetylglutamate kinase (NAGK, EC 2.7.2.8., ArgB) [9], the next enzyme in the pathway. The recent explosion of genomic data has enabled us to find other organisms endowed with the *argH(A) *fusion as well as many instances where a sequence homologous to *arg(A) *is not fused to *argH*. Moreover, as this work was in progress, a new functional ArgA protein (gene Rv2747) has been characterized in *Mycobacterium tuberculosis *[10]. Interestingly, the cognate gene is found to be homologous to *Moritella arg(A)*. These findings have important implications regarding the formation of acetylated intermediates in arginine biosynthesis and the evolution of the cognate enzymes.

## Results and Discussion

### Occurrence of the argH(A) gene

While studying arginine biosynthetic genes in two vibrio-like strains (later characterized as novel psychropiezophilic *Moritella *species *M. abyssi *and *M. profunda *[8]) we found most *arg *genes clustered into a divergent operon-like structure composed of two wings: a leftward one comprising the sole *argE *gene and a rightward one *argCBFGH(A) *where *argH *is extended by a ± 170-long codon stretch, in translational continuity. This extension was shown to complement an *E. coli *auxotroph deficient in NAGS [7], demonstrating that it encodes an ArgA-like activity (EC 2.3.1.1).

**Figure 3:**
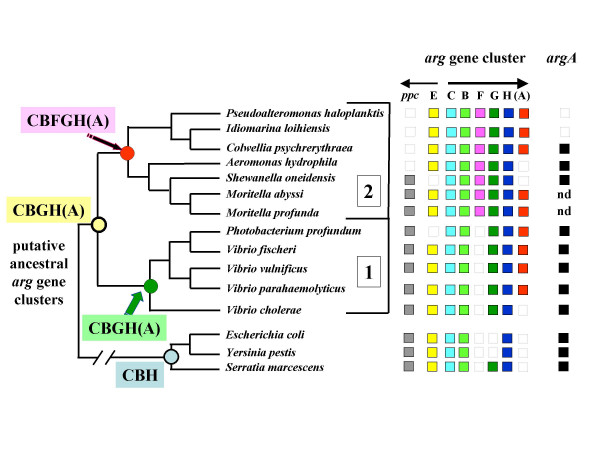
**Phylogenetic relationships of species harbouring ArgH(A) fusions**. A simplified version of the 16S rRNA phylogenetic tree of *Alteromonas*- and *Vibrio*-like bacteria reconstructed by Ivanova et al.[11] has been drawn and enriched with the following information. On the right-hand side, available genomic information concerning *arg *genes; *ppc *refers to the gene for phosphoenolpyruvate carboxylase, adjacent to *argE *in many of these organisms; nd: not determined. On the left-hand side, the putative content of the ancestral *arg *gene clusters are indicated for each deep node of this tree.

We searched for the presence of the argH(A) fusion gene homologues in completely sequenced microbial genomes. Blast analyses identified nine bacteria that are phylogenetically related to *Moritella*. This is shown in Fig. 3, which combines a simplified and partial version of an extensive 16S rRNA tree for *Alteromonas *and *Vibrio*-like bacteria [11] with genomic information about the structure of the *arg *gene cluster. Most of these organisms display the divergent pattern characteristic of vibrio-like and enteric bacteria, which contrasts with the more scattered pattern encountered in other branches of the gamma proteobacteria [2,7]. There are differences: the shorter version clusters *argE *with *argCBH*, as in *E. coli *and *Yersinia pestis*, whereas the right wing is longer in several other bacteria. Thus, *argCBGH *was probably present already in the common ancestor to clades 1 and 2, and *argCBFGH *in the ancestor to clade 2. Note that *argE *is not clustered with other *arg *genes in *Shewanella oneidensis *and *Photobacterium profundum*.

In conclusion:

(**i**) *argH(A) *appears to be restricted to this particular group of marine Bacteria. It is noticeable that *V. cholerae*, which is not a marine organism, does not have *argH(A) *whereas the three marine *Vibrio *species do: *V. fischeri, V. vulnificus *and *V. parahaemolyticus*. This pattern suggests that the presence of *argH(A) *is the result of orthologous transfer in diverging lines of descent sharing a common habitat (the sea), perhaps accompanied by some lateral transfer among them as discussed below. Fig 3 suggests that the *H(A) *fusion occurred in an ancestor common to clades 1 and 2, but this can not be ascertained without an extensive search among the numerous members of this group, in particular among different *Idiomarina *and *Pseudoalteromonas *species, as well as in the genera branching early on this tree.

(ii) No correlation appears to exist between the presence of *argH(A) *and either psychrophily or piezophily. Indeed *argH(A) *was found among mesophiles (*V. fischeri, V. vulnificus, V. parahaemolyticus)*, psychrophiles (*Colwiella psychrerythraea, Pseudoalteromonas haloplanktis*), psychro-piezophiles (*P. profundum*, *M. abyssi, M. profunda*) and meso-piezophiles (*Idiomarina loihiensis*). As the cardinal temperatures and hydrostatic pressures of these closely related organisms actually overlap, lateral transfer among them seems feasible even if the reality of the phenomenon is beyond experimental proof.

(iii) Sequences homologous to the full *E. coli argA *gene were found in several of the organisms harboring the *H(A) *fusion but not in *I. loihiensis *and *P. haloplanktis *(for *M. abyssi *and *M. profunda*, their genomes have not yet been sequenced). In *I. loihiensis *and *P. haloplanktis*, the *(A) *sequence therefore does not appear to be functionally redundant, which in turn suggests that these organisms, which can grow in absence of arginine [12,13], depend exclusively on domain *(A) *for the first step of arginine biosynthesis.

### Origin of argH(A)

The data suggest that *argH(A) *results from a fusion that occurred between *argH *and a gene coding for an acetyltransferase able to acetylate L-glutamate in the N- position. The fusion could have been selected for in an organism devoid of a canonical NAGS, such as *I. loihiensis *and *P. haloplanktis*, perhaps as the result of gene loss, or it may reflect a more primordial event. Interestingly, in *I. loihiensis*, the genes of the *argCBFGH(A) *cluster are tightly coupled, either overlapping by 3 nt (*argC *and *B*, *argF *and *G*), separated by 3 nt (*argB *and *F*) or by 4 (*argG *and *H*). This arrangement suggests that, at the time the fusion originated, the capacity to derepress the recruited acetyltransferase from the rightward promoter of the operon may have been essential and actually explains why this fusion took place. In keeping with this hypothesis, the genome of *I. loihiensis and P. haloplanktis do *contain a sequence homologous to the *E. coli argR *regulatory gene.

### Occurrence of arg(A)-like sequences with putative function in other organisms

**Figure 4:**
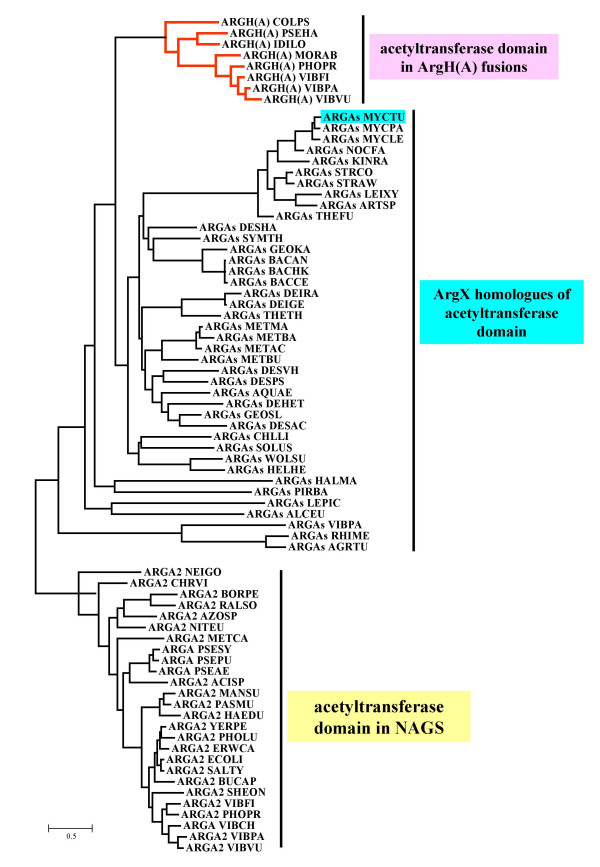
**Phylogenetic tree of the *arg(A) *homologues**. The sequences have been collected, multiply aligned and used to reconstruct an evolutionary tree as described in Methods. The tree has been rooted using the acetyltransferase domain of NAGS sequences as an outgroup (subtree indicated with yellow color). The two other subtrees corresponding to the Arg(A) and ArgX sequences are indicated with pink and blue colors, respectively. Species names are according to SwissProt conventions (the detailed list is shown in Table 1).

If there were an N-acetyltransferase gene susceptible to recruitment in a bacterium related to *Idiomarina*, it would probably be present in many genomes. In order to test this hypothesis we employed a two-step strategy. First, we screened prokaryotes for the presence of acetyltransferase genes similar to the *arg(A) *sequence of *M. abyssi *(see Methods). Fourty-four completely sequenced genomes have homologous genes that were annotated as coding for either a hypothetical protein or a putative acetyltransferase. These *argX *genes were aligned with the *arg(A) *sequences and with the homologous acetyltransferase domain of NAGS. A phylogenetic tree was computed from this alignment using a maximum likelihood approach and rooted with the acetyltransferase domain of classical NAGS enzymes. Fig. 4 shows that the *arg(A) *sequences form a monophyletic group which share a common ancestor with a large group containing the short NAGS version recently found in *M. tuberculosis *[10]. This tree also shows the complex relationships between the different paralogous *argA*-like sequences. For instance, *V. parahaemolyticus *contains three related sequences: the fused *arg(A)*, an *argX *that is a remote paralogue to this *arg(A)*, and the classical *argA *(NAGS). It is not clear what are the respective roles of these different paralogues in cell metabolism.

Furthermore, we fused these *arg(A)*-like sequences *in silico *with *argH *sequences from the same organism in order to build so-called *argHX *sequences. Since *argH *is in the the last step of the pathway, and the *(A) *determinant in the first one, we focused the search on organisms presumed to possess the whole pathway and, took into account any structural and/or functional significance of the association of the two determinants. All 26 species found using this approach form a homogeneous group presenting the following features: (i) they do not possess a multidomain homologue of a NAGS protein, (ii) they contain an ornithine acetyltransferase (ArgJ) and (iii) they lack an acetylornithinase gene (ArgE). Thus, these 26 species should use an alternative to NAGS in order to acetylate glutamate and are presumed to recycle the transfer of the acetyl group from acetylornithine to glutamate. In contrast, the species retrieved in the first step of the screening, before implementing the virtual fusion approach, form a wider and less homogeneous group (Fig. 4), where NAGS and/or ArgE can be found. This suggests that the virtual fusion approach identifies a functionally significant group.

**Figure 5:**
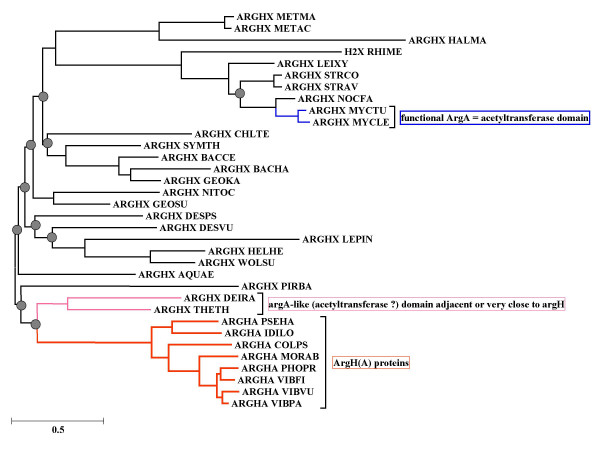
**Phylogenetic tree of the ArgH(A) and ArgHX fusions**. The sequences have been collected, multiply aligned and used to reconstruct an evolutionary tree as described in Methods. The grey circles indicate the bootstrap values for the deep nodes which are less than 60%. Species names are according to SwissProt conventions (the detailed list is shown in Table 1).

The different, virtual *argHX *sequences were further aligned with their homologues *argH(A) *and an evolutionary tree was reconstructed using a maximum likelihood approach (Fig 5). Note that the large size of *argH *(more than 420 residues) and its relatively high degree of conservation increase the overall similarities between the *argH(A) *fusions. This could contribute to the topological differences between the trees in Figs. 4 and 5. The phylogenetic analysis of the fusion approach displays intriguing aspects:

i. The *argH(A) *fusions form a monophyletic group branching close to homologous sequences present in the genomes of both *Thermus *and *Deinococcus*, two phylogenetically related organisms. The two groups join at a node position where the bootstrap value is less than 60 %, but it is remarkable that the *Thermus *and *Deinococcus *sequences (annotated as homologues of *argH *and a putative acetyltransferase gene), are actually adjacent, suggesting that together they play the role of a functional analogue of *argH(A). T. thermophilus argH *(which overlaps *argG *by 10 nt at the proximal end) and the putative acetyltransferase gene are separated by only 2 nt, strongly suggesting that the *Thermus arg(A)-*like sequence is part of an *argGH(A) *operon in the arginine regulon of this organism [14]. In *D. radiodurans *the situation is similar but more complex: no less than three putative acetyltransferase genes are adjacent to *argG *(the first one separated by only 2 nt) while *argH *is very closely linked to another three putative acetyltransferase genes; of these three it is the last one (Q9RWI5_DEIRA Hypothetical protein DR0683) that is retrieved by our homology search.

ii. The other part of the tree contains various prokaryotic species including mesophilic Archaea. Clustering with these Archaea, we note a clade of Actinobacteria including *M. tuberculosis*. This is highly significant since the *M. tuberculosis *sequence was shown tocode for an enzyme whose functional characterization was reported while this paper was being prepared for publication: it displays acetylglutamate synthetase activity [10] and is required for the growth of its host as shown by previous high-density mutagenesis [15].

An essential question is whether any other of these 26 prokaryotes harboring an *arg(A)-*like sequence actually depend on it for acetylation of glutamate *in vivo*. We know (see above) that the complete sequence of their genome lacks a classical NAGS. The presumption would be even stronger if these species possessed a monofunctional OAT, and were thus unable to acetylate glutamate with acetyl-CoA [5,16,17]. Currently, *T. thermophilus *and *Streptomyces coelicolor *fulfill this second criterium [ibid, [18]]. The (A)-like sequence of *T. thermophilus *moreover appears co-regulated with the *argGH *cluster. It is not known whether *M. tuberculosis *OAT is monofunctional, but the fact that the *arg(A)-*like sequence of this organism was shown to be essential by transposon-mediated inactivation [15] actually suggests that it is. Since it is not yet possible to predict *in silico *whether a particular OAT is bifunctional [19], biochemical evidence is needed to decide which of the other microorganisms actually depend on their *arg(A)-*like sequence for arginine biosynthesis.

### Comparative analysis of putative glutamate N-acetyltransferases

**Figure 6:**
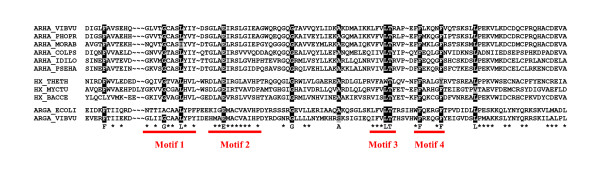
**Conserved motifs in putative glutamate N-acetyltransferases**. The homologues to the (A) domain of ArgH(A) of *M. abyssi *and belonging to six ArgH(A), three ArgHX and two ArgA proteins have been multiply aligned and edited in BioEdit software [32]. A consensus sequence has been computed. Very strongly conserved residues(letters) and moderately conserved ones (stars) are indicated. The four motifs containing the residues identified by comparative analysis of N-acetyltransferases which use acetylCoA as donor of the acetyl group [20] and references therein] are underlined by thick lines and numbered. Species names are according to SwissProt conventions.

The polypeptides encoded by the fused *arg(A) *sequences, their X homologues and the C-terminal domain of ArgA (NAGS) belong to the vast superfamily of GNC5-N-acetyltransferases (GNAT), all using acetylCoA as common acetyl donor. Multiple alignment of these three categories of homologous sequences (Fig. 6) indicates good conservation of the four motifs which were previously identified by comparing numerous members of this superfamily [[20,21] and references therein]. Most GNAT acetyltransferases, including NAGS, proceed by a sequential mechanism, *i.e*. form a ternary complex of enzyme, acetylCoA and specific substrate, and not by a ping-pong mechanism involving the formation of an acetylthioenzyme intermediate between acetylCoA and an active cysteine [21,22]. In keeping with these observations, there is no strictly conserved cysteine among the sequences reported in Fig.6. More accurate prediction of catalytic residues is difficult without a NAGS 3D-structure, in particular as regards the glutamate binding site. The fact that *Moritella arg(A) *complements an NAGS-inactivated mutant of *E. coli *[7] and that pure *M. tuberculosis *ArgA acetylates glutamate *in vitro *[10] confirms that such a site is present in these proteins even though their sequence corresponds only to the C-terminal domain of classical NAGS. It is possible that the short version of NAGS has to be associated with another protein in order to bind glutamate efficiently, which makes attempts at site prediction premature without further empirical testing.

## Conclusion

The discovery of a novel type of biosynthetic *arg *locus, coding for a classical argininosuccinase ArgH fused with a putative N-acetyltransferase able to complement an *argA *deficiency was extended by genomic analysis to a group of phylogenetically and ecologically related marine gamma proteobacteria. The case of *I. loihiensis *[12] and *P. haloplanktis *[23] is particularly significant from the functional point of view since the cognate genomes do not appear to contain a genuine *argA *sequence and the organisms are nevertheless arginine-independent, indicating that they depend on Arg(A) for arginine biosynthesis. Note there is widespread occurrence of sequences homologous to *arg(A) *in organisms lacking a classical NAGS (Fig. 5), including instances (*Thermus *and *Deinococcus*) where the sequence is adjacent-to, and coexpressed with *argH*. Moreover *T. thermophilus *and *S. coelicolor *which do not possess an OAT able to acetylate glutamate with acetylCoA, most probably depend on their *arg(A) *homologue for arginine biosynthesis.

The gene-enzyme relationship for the first committed step of arginine biosynthesis must now be considered in a new perspective. Several alternatives can be recognized:

i. The classical NAGS originally found in *E. coli *and *Pseudomonas *has two domains: an N-terminal one, with a carbamate kinase fold, displays extensive similarity with acetylglutamate kinase (NAGK), while the C-terminal one contains an N-acetyltransferase fold. This classical NAGS may occur in organisms with an acetylornithinase (ArgE) or an ornithine acetyltransferase (ArgJ), two situations epitomized by *E. coli *and *P. aeruginosa*. In *P. aeruginosa*, where ArgJ only recycles the acetylgroup from acetylornithine, NAGS fulfils an anaplerotic, but essential function, priming arginine biosynthesis with the acetyl group from acetyl-CoA [1,2].

ii. In *B. stearothermophilus *and *T. neapolitana *the ArgJ (OAT) protein is bifunctional: not only does it recycle the acetyl group, but it also catalyzes the first step (EC 2.3.1.1). Early data concerning expression of *B. subtilis *genes in *E. coli *[24], reinterpreted after sequencing of the cognate DNA show that this bacterium also has a bifunctional OAT. In principle, such organisms do not need a NAGS, an assumption corroborated by the actual lack of a NAGS gene in the genomes of their close relatives, *B. subtilis *and *T. maritima*. It is worth noting that bifunctional OAT does not show recognizable similarity with NAGS despite the fact that they catalyze the same reaction [5,19].

iii. In eukaryotes, reaction EC 2.3.1.1 is carried out by different kinds of multidomain proteins that are not homologous. In fungi, NAGS activity actually requires association of NAGS with NAGK [25,26] but contrary to what occurs in prokaryotes, there is no significant similarity between NAGS and NAGK (the carbamate kinase fold). On the other hand, mammalian NAGS is similar to the bimodular *E. coli *NAGS: it possesses a carbamate kinase fold and is able to complement an *argA *mutant of *E. coli *[27].

iv. The discovery of the *in vivo *active *Moritella arg(A) *sequence fused to the *argH *gene, and the detection of several homologous *argH(A) *sequences among marine Proteobacteria including species devoid of NAGS (*I. loihiensis, P. haloplanktis*) indicates that reaction EC 2.3.1.1 can be catalyzed by a short version of NAGS corresponding to the C-terminal domain of the bimodular NAGS. Furthermore, a number of previously uncharacterized acetyltransferases from different prokaryotes are homologous to this shorter version and the recent biochemical characterization of one of them in *M. tuberculosis *strongly suggests that many organisms rely on this monodomain form of NAGS to synthesize acetylglutamate.

**Figure 7:**
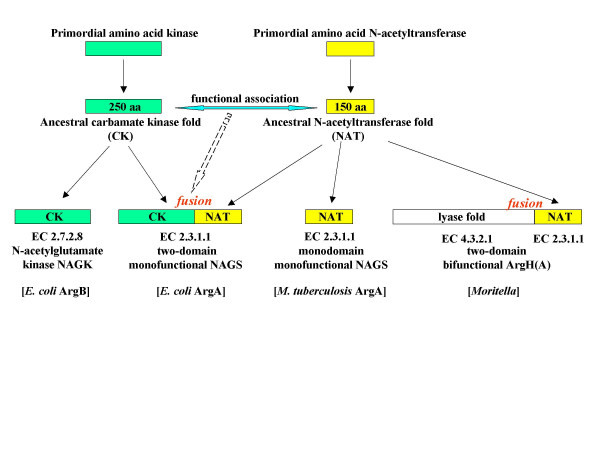
**Evolution of glutamate acetylation from a primordial N- acetyltransferase**. The recruitment of a primordial N-acetyltransferase for L-glutamate acetylation in the first step of arginine biosynthesis has been made according to at least three different evolutionary ways. The events of gene duplication and gene fusion that allowed evolution toward either the two-domain N-acetylglutamate synthase (EC 2.3.1.1) or the bifunctional *argH(A) *fusion are schematized. This scheme does not specify whether the *argH(A) *fusion arose in an organism originally devoid of NAGS or in an organism having lost NAGS. The yellow domains in contemporary proteins (bottom line) are bearing the ArgA activity (EC 2.3.1.1). The contemporary proteins that have been experimentally studied are indicated between brackets.

Perhaps this monodomain Arg(A)-like sequence is a primordial enzyme originally recruited from an ancient pool of N-acetyltransferases (see Fig. 7 for a possible evolutionary scheme). The classical, two-domain NAGS may have arisen under selection for an efficient glutamate-binding site as the result of a fusion between this protein and an N-terminal domain which is also found in NAGK. It must be noted that the monodomain *M. tuberculosis *NAGS has a considerably higher Km for glutamate than the *E. coli *two-domain one [10]. Association with NAGK, perhaps by providing an efficient glutamate-binding site, may alleviate this kinetic shortcoming. This association may have preceded the advent of the classical NAGS by fusion of the two domains into a single protein (see Fig. 7). In organisms with an *argH(A) *fusion, physical association between ArgH and Arg(A) might enhance Arg(A) activity or stability and perhaps render the enzyme sensitive to arginine as a feedback inhibitor, since arginine is a product of the reaction catalyzed by ArgH. At any rate, a capital effect of the fusion is probably to have brought *arg(A) *under regulated expression by arginine. The presence of a classical NAGS (gene *argA*) along with the *argH(A) *sequence in some organisms (vibrio-like, enterics) having an AO (ArgE) but no OAT (ArgJ) could be explained by the fact that such organisms are unable to recycle the acetyl group and therefore require a larger flow of acetylglutamate. This hypothesis can be tested by comparing such organisms with *Idiomarina *and *Pseudoalteromonas *(where *argA*-encoded NAGS is absent) for growth and glutamate acetylation kinetics.

Due to a lack of biochemical evidence for OAT, we do not know which of the two isoforms, mono or bifunctional, is the more widespread and possibly the primordial one. One possibility is that an arginine pathway using a bifunctional OAT and devoid of NAGS is the most ancestral version of the biosynthesis and that the various forms of NAGS we have been discussing appeared under selection after a mutation transformed a bifunctional OAT into a monofunctional enzyme. The acetylornithinase found in enteric and vibrio- like bacteria may have emerged after loss of such an OAT (7).

The alternatives emphasized in the present survey do not appear to exhaust the variety of solutions implemented in nature for the N-acetylation of glutamate. Archaea such as *M. jannaschii *have a monofunctional OAT [5] but no homologues of either mono- or two-domain ArgA could be revealed by our investigations. It is therefore possible that in such organisms the reaction EC 2.3.1.1 is carried out by yet another protein. In this respect, organisms such as *Xanthomonas *appear rather puzzling. In the entirely sequenced genomes of the three available species, a gene encoding a short protein similar to an acetyltransferase has been annotated *argA*. However, although this gene is clearly inside the *arg *cluster (between *argC *and *argB*) it does not appear as homologous to any of the known mono- or two-domain ArgA proteins and it is absent from the closely related *Xylella *species. It might therefore represent a new acetylglutamate synthetase or been incorrectly annotated.

In conclusion, our concept of the gene-enzyme relationships in arginine biosynthesis is undergoing a drastic revision among prokaryotes. Far from being universal, the patterns of acetylation of the intermediates may differ in phyla and even within the same phylum. The basic acetylation strategy that segregates arginine and proline precursors in different pathways is not brought into question, but the identity and the origin of the enzymes responsible for glutamate acetylation appears to betray extensive "natural tinkering" [28]. Further phylogenetic analysis and the structural characterization of the cognate proteins will hopefully shed some light on the evolution of this crucial metabolic step.

## Methods

### Identifying all sequences homologous to the Arg(A) domain

In a first step, the *M. abyssi *sequence [7] was used as a query to collect the ArgH(A) homologous sequences from the last version (September 2005) of Uniprot (SwissProt and TREMBL) using the Blast facilities of the ExPaSy server [29] and the following criteria : ranking among the best E-values and aligning along the full length (around 620 aa) of the query. Note that several of the found homologues have been incorrectly annotated as "bifunctional protein ArgH" (*V. parahaemolyticus*), "argininosuccinate lyase" (*I. loihiensis*, *V. vulnificus *YJ016), "amino-acid acetyltransferase" (*V. fischeri*), N-acetylglutamate synthase (*V. vulnificus *CMCP6). These ArgH(A) sequences were immediately followed in the Blast outfile by the whole set of ArgH proteins (around 450 residues long). Preliminary sequence data was obtained from [30] in the case of *Aeromonas hydrophila*.

In a second step, the sequence of the domain (A) of the ArgH(A) protein of *M. abyssi *(KAVGTFAVTEKHNQVTGCASIYVYDTGLAELRSLGIEPGYQGGGQGKAVVEYMLRKAEQMAIQKVFVLTRVPEFFMKLGFRSTSKSMLPEKVLKDCDMCPRQHACDEVALEFKLNVVGQTINLKAEKLAS) was further used do detect (A) homologues. We first identified a list of short (150–180 residues) proteins generally annotated as putative acetyltransferases and, in a second, more distant wave, the acetyltransferase domain of the canonical ArgA (NAGS) such as that of *E. coli*.

### Reconstructing phylogenetic trees

All the (A) homologous sequences were multiply aligned using ClustalX [31]. The *arg(A)*-like sequences were virtually fused *in silico *with *argH *sequences from the same organism in order to build so-called *argHX *sequences. The ArgH(A) and ArgHX sequences were further mutiply aligned. Both automatic alignments were manually improved using the BioEdit software [32], saved in PHYLIP format and further used to reconstruct phylogenetic trees applying a two-step maximum likelihood approach as follows. After computing a BIONJ [33] distance tree using the Dayhoff model of evolution [34], program Phyml [35] was employed to refine this initial distance tree and to optimize its topology using a discrete-gamma model to accommodate rate variation among sites. The shape parameter alpha of the gamma distribution was estimated as described in [36] and found to be 3.74, the proportion of invariant sites being 0.065. The confidence limits for each node were further estimated using the non-parametric bootstrap approach of the Phyml program with 100 computed data sets.

## Authors' contributions

YX and NG were at the origin of this work that extends their initial discovery. NG and BL designed the approach to hunt for relevant homologous sequences and YX and BL made the phylogenetic studies. NG and BL wrote the manuscript that has been read and approved by the three authors.

## Figures and Tables

**Figure 2:**
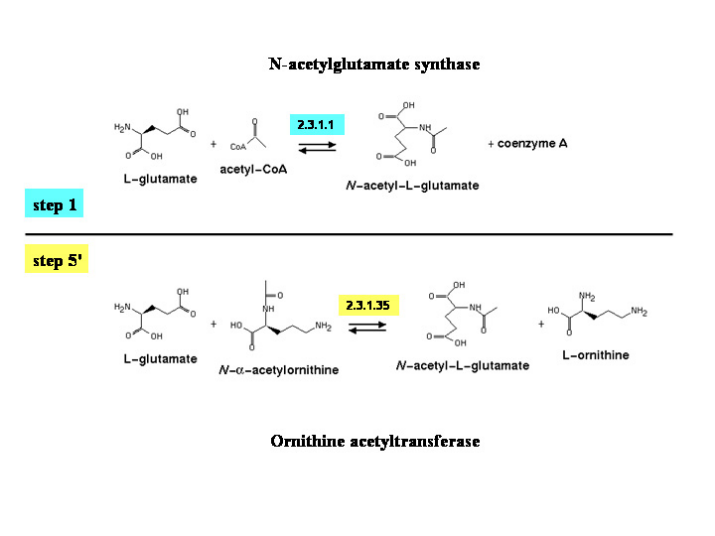
**Reactions involving L-glutamate.  **This figure has been drawn using the tools available at Metacyc [37].

**Table 1:** Full names of in alphabetical order species used in phylogenetic studies

**Abbreviation**	**Complete species name**^**a**^
AGRTU	*Agrobacterium tumefaciens*
AQUAE	*Aquifex aeolicus*
ARTSP	*Arthrobacter species*
BACCE	*Bacillus cereus*
BACHA	*Bacillus halodurans*
BACTH	*Bacillus thuringiensis*
CHLLI	*Chlorobium limicola*
CHLTE	*Chlorobium tepidum*
DEHET	*Dehalococcoides ethenogenes*
DEIGE	*Deinococcus geothermalis*
DEIRA	*Deinococcus radiodurans*
DESHA	*Desulfitobacterium hafniense*
DESPS	*Desulfotalea psychrophila*
DESVH	*Desulfovibrio vulgaris*
DESAC	*Desulfuromonas acetoxidans*
GEOKA	*Geobacillus kaustophilus*
GEOME	*Geobacter metallireducens*
GEOSL	*Geobacter sulfurreducens*
HALMA	*Haloarcula marismortui*
HELHE	*Helicobacter hepaticus*
KINRA	*Kineococcus radiotolerans*
LEIXY	*Leifsonia xyli*
LEPIN	*Leptospira interrogans*
METBU	*Methanococcoides burtonii*
METAC	*Methanosarcina acetivorans*
METBA	*Methanosarcina barkeri*
METMA	*Methanosarcina mazei*
MYCLE	*Mycobacterium leprae*
MYCPA	*Mycobacterium paratuberculosis*
MYCTU	*Mycobacterium tuberculosis*
NOCFA	*Nocardia farcinica*
ALCEU	*Alcaligenes eutrophus*
RHIME	*Sinorhizobium meliloti*
PIRBA	*Pirellula baltica*
SOLUS	*Solibacter usitatus*
STRAV	*Streptomyces avermitilis*
STRCO	*Streptomyces coelicolor*
SYMTH	*Symbiobacterium thermophilum*
THEFU	*Thermobifida fusca*
THETH	*Thermus thermophilus*
VIBPA	*Vibrio parahaemolyticus*
WOLSU	*Wolinella succinogenes*

^a ^Names of species used in the alignment of ArgHX sequences are listed as found in the phylogenetic tree shown on Figs. 3 and 4.
